# Properties of Particle Size Distribution from Milled White Nixtamalized Corn Kernels as a Function of Steeping Time

**DOI:** 10.1155/2016/6724047

**Published:** 2016-06-08

**Authors:** J. L. Fernández-Muñoz, M. Zapata-Torrez, A. Márquez-Herrera, F. Sánchez-Sinencio, J. G. Mendoza-Álvarez, M. Meléndez-Lira, O. Zelaya-Ángel

**Affiliations:** ^1^Instituto Politécnico Nacional, CICATA LEGARIA, Calzada Legaria No. 694, Colonia Irrigación, 11500 México, DF, Mexico; ^2^Departamento de Ingeniería Agrícola, DICIVA, Universidad de Guanajuato, Campus Irapuato-Salamanca, Ex Hacienda el Copal km 9, Carretera Irapuato-Silao, 36500 Irapuato, GTO, Mexico; ^3^Departamento de Física, Centro de Investigación y de Estudios Avanzados del Instituto Politécnico Nacional, Apartado Postal 14-740, 07000 México, DF, Mexico

## Abstract

This paper focuses on the particle size distribution (PSD) changes during nixtamalized corn kernels (NCK) as a function of the steeping time (ST). The process to obtain powder or corn flour from NCK was as follows: (i) the NCK with different STs were wet-milled in a stone mill, (ii) dehydrated by a Flash type dryer, and (iii) pulverized with a hammer mill and sieved with a 20 mesh. The powder was characterized by measuring the PSD percentage, calcium percentage (CP), peak viscosity at 90°C (PV), and crystallinity percentage (CP). The PSD of the powder as a function of ST was determined by sieving in Ro-TAP equipment. By sieving, five fractions of powder were obtained employing meshes 30, 40, 60, 80, and 100. The final weight of the PSD obtained from the sieving process follows a Gaussian profile with the maximum corresponding to the average particle obtained with mesh 60. The calcium percentage as a function of ST follows a behavior similar to the weight of the PSD. The study of crystallinity versus the mesh number shows that it decreases for smaller mesh number. A similar behavior is observed as steeping time increases, except around ST = 8 h where the gelatinization of starch is observed. The trend of increasing viscosity values of the powder samples occurs when increasing ST and decreasing particle size. The ST significantly changes the crystallinity and viscosity values of the powder and, in both cases, a minimum value is observed in the region 7–9 h. The experimental results show that the viscosity increases (decreases) if the particle size decreases (increases).

## 1. Introduction

Maize (*Zea mays* L.) is the third most important cereal grain for human consumption in the world after wheat and rice and is a staple food in many regions. It is estimated that, in 2012, the global production of maize was around 900 million tons, with the United States of America, China, Brazil, and Argentina being the top maize-producing countries [1]. Maize is processed to make food and feed ingredients using physical or chemical processing methods. Currently, corn flour products are employed in around 45% of the Mexican tortilla market. The tortilla industry represents approximately one-fifth of the overall maize market of Mexico, with an annual production estimated at 11.5 million tons, producing sales valued at 7 billion dollars [2].

The physicochemical state of different PSD fractions is considered to be an important criterion for maize flour used in the production of tortillas or derived products [3]. It has been found that, employing a single alkaline thermal treatment and ST, corn tortillas preparation requires a particular formulation of fine, medium, and coarse particles. The fine particle size is important to develop flexibility and cohesiveness, whereas chips require coarse particles to promote crispness after frying [4]. When the steeping time increases, the crystallinity and viscosity properties of the PSD are more homogeneous allowing one to obtain a better tortilla product [5]. Improvement in the quality of the tortilla products employing more than one ST has been corroborated in three industrial plants in Mexico [6].

Physicochemical, morphological, and apparent viscosity changes in corn flour samples prepared using high quality protein maize were determined as a function of ST for different PSD [7]. The calcium percentage (CP) content in the particle size does not follow a linear relationship with the steeping time, in agreement with published results [8]. An analysis of pasting characteristics based on the profile curves was done to study the apparent viscosity of high quality protein maize; a dependence of viscosity on the particle size was found stablishing the fact that the Rapid Visco Analyser (RVA) technique can describe maize hardness [7, 9].

In general, the shape of RVA profiles depends on maize hardness and the aggregation of flour particles the viscosity peak and breakdown point increased for soft corn and decreased for hard maize. In a study of industrial corn flour obtained employing unfractionated and sieved corn flour using meshes 40, 60, and 70, the peak viscosity and breakdown point were not observed  [4]. In the preparation of tortillas it is important to determine the peak viscosity and breakdown point of the PSD to obtain the optimal values of quality, shelf life, and yield. Thus, the main objective of the present is to characterize corn flour obtained using meshes 30, 40, 60, 80, and 100 through viscosity at 90°C, PSD, CP, and crystallinity percentages as a function of the steeping time. To the best of our knowledge there are no reports on the effect of the increase of calcium percentage, ST, and coarse size of particles on peak viscosity and breakdown point.

## 2. Materials and Methods 

### 2.1. Samples

The following procedure was used to prepare the corn flour samples: 6 L of water, previously heated to a temperature of 92°C, was added to 3 kg of commercial white dent maize (Toluca region) (1 : 2 maize-water ratio). Immediately 60 g of Ca(OH)_2_ was added (MERCK nutritional grade reactive powder). This mixture was cooked for 40 min at 92°C. After the cooking process, the maize was steeping in the cooking water for 0, 3, 5, 7, 8, 9, 11, 13, 15, and 24 h, taking samples at the predetermined ST. Then the cooking water (nejayote) of each sample was drained off, and the nixtamal was washed twice with water for 2 minutes. Finally, the washed nixtamal was grounded in a stone mill (FUMASA, US-25 model) and then dehydrated in a Flash type dryer. To turn it into corn flour the material from the Flash type dryer was pulverized with in a hammer mill employing a 20 mesh.

## 3. Characterization Techniques

### 3.1. Measurements PSD

The PSD of the corn flour was measured using Ro-TAP equipment with a set of meshes: 30 (595 *μ*m), 40 (400 *μ*m), 60 (250 *μ*m), 80 (177 *μ*m), and 100 (149 *μ*m). The sieving of the corn flour determined the PSD groups. The sieving procedure was done during 15 minutes employing 150 g of corn flour as reported in a previous work [5]. The fractions retained in each mesh were separated and weighed. The PSD fractionation was replicated three times.

### 3.2. Calcium Percentage (CP)

The CP was obtained by atomic absorption spectroscopy ([10], Analyst 300, Perkin Elmer). The spectrometer parameters used were 12 psi dry air pressure, 70 psi acetylene pressure, lamp with a wavelength of 422.7 nm operated at a current of 10 mA, and a slit aperture of 0.7 nm.

### 3.3. Rapid Visco Analyser (RVA) Measurements

Rheological properties of PSD samples were measured by the Rapid Visco Analyser (RVA-Series, Newport Scientific). The relative viscosities of the PSD water suspensions of corn flour dough were determined using a pasting viscometer. Suspensions of corn flour produced at different ST were prepared to determine RVA profile. The total time to obtain the RVA graphs was 15 min. They were obtained by raising the temperature of the corn flour suspension from 50 to 90°C. The samples were maintained at the higher temperature during 5 min, and then the temperature was decreased at the same rate at which it had increased. For each sample, 4 g of corn flour, with a humidity of 12%, and 24 milliliters of distilled water were used.

### 3.4. X-Ray Diffraction

The corn flour samples were compressed and placed in a sample holder. The X-ray diffraction study was made from 4° to 30° with a step of 0.05°. A monochromatic CuK_*α*_ radiation (wavelength *λ* = 1.5406 Å) was employed, on a Siemens D5000 diffractometer operating at 35 KV and 15 mA. The relative percentage values for the crystallinity of the PSD from corn flour were calculated using software integrated into the measurement system.

## 4. Results and Discussion

### 4.1. PSD Measurements

**Figure 1:**
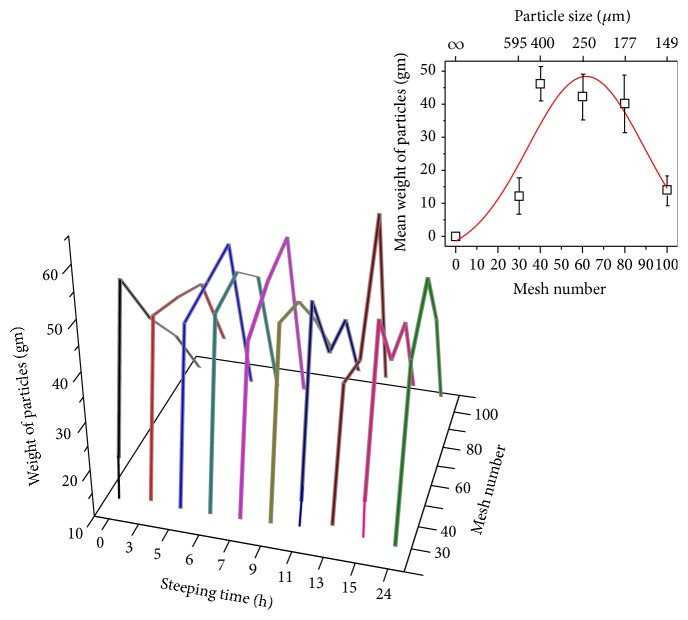
The weight of PSD (W-PSD) as a function of the mesh numbers for all the steeping times (ST) values studied. The inset illustrates the mean W-PSD value versus the mesh number averaged on the five mesh screens selected for the analysis. The red line is a fitting by using a Gaussian function.

**Figure 2:**
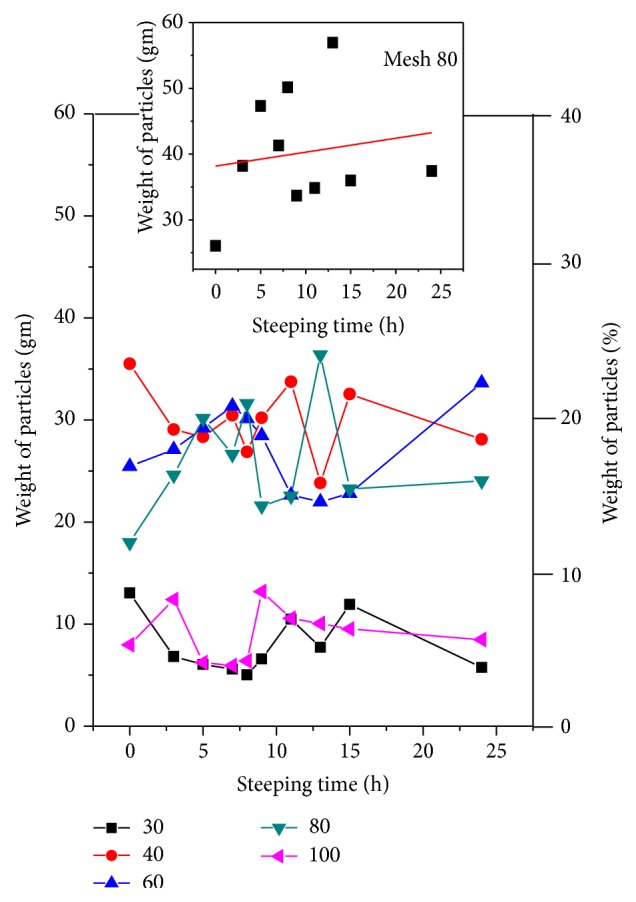
W-PSD versus ST for the five meshes chosen. The inset displays the linear fitting as a first approximation of W-PSD against ST, for the mesh 80, to measure a mean slope of experimental points.

The weight of the set of PSDs (W-PSD) as a function of the mesh number and ST was determined (fixes the maximum particle size in the PSDs) for each of the ten STs. As shown in Figure 1 the PSD graphs are characterized by having the aspect of a zigzag-line arch. The small top panel displays the averaged values of the W-PSD, for each of the different STs, against the mesh number (maximum value of the particle size in each mesh). The red line is a fitting to the experimental data using a Gaussian curve to represent the corn flour milling process. In general, it represents a normal distribution of particles [11]. The PSD of the corn flour samples, for each one of the mesh numbers employed, as a function of ST is displayed in Figure 2 (the percent of the particles is indicated in the vertical right-axis). Each of these fifty experimental values represents the average of three measurements per sample. Checking the results shown in this figure, one can distinguish two PSD set groups: the first group retained into the 30 and 100 mesh screens varied between 4 and 14%, and the second one retained in the 40, 60, and 80 mesh screens varied from 80 to 90%. The PSD versus ST behavior reflects the distribution suggested in the inset of Figure 1. The inset in Figure 2 illustrates a linear fitting of several PSD values versus ST. This fitting was carried out with the purpose to catch sight of a tendency of the like-saw-tooth-lines when ST increases. The average value of the slopes of the five lines has a positive average value which indicates that the weight of the particles increases during ST. This fact could indicate that the corn flour weight kept on the meshes augments as the ST augments as a result of the more water and calcium diffusion during nixtamalization and the milling process.

### 4.2. CP Measurements

**Figure 3:**
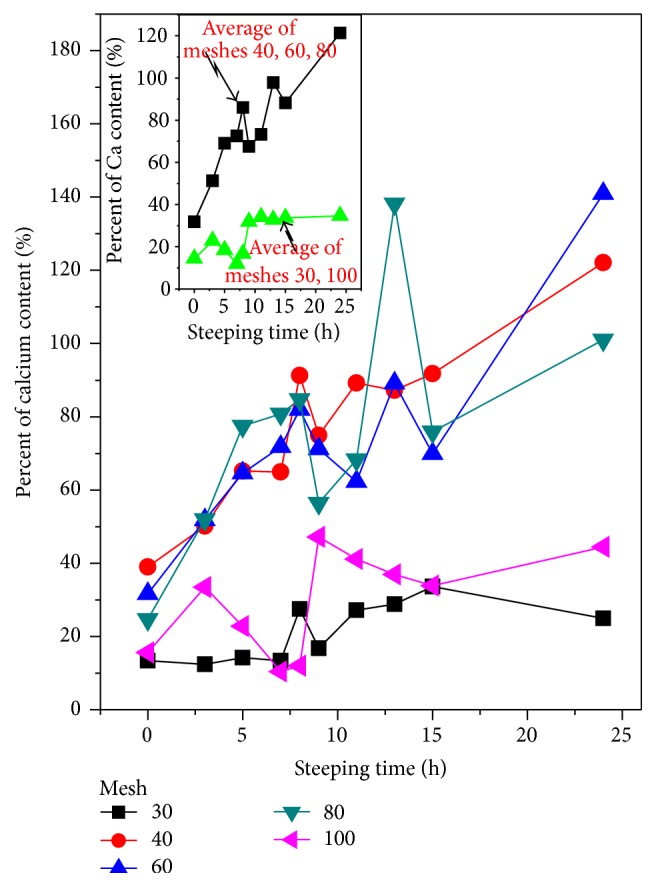
Calcium percentage (CP), absorbed by the PSD from the alkaline solution, as a function of ST for all the meshes used. The inset exhibits the mean CP averaged on 40, 60, and 80 (black squares) and 30 and 100 (green triangles), as a function of ST.

Figure 3 exhibits the CP in PSD of the corn flour as a function of the ST. These experimental values represent the average of three measurements per sample. The experimental points are scattered in a similar distribution of W-PSD in Figure 1. This fact suggests that the CP in the particles is proportional to the PSD weight. On the other side, as can be seen in Figure 3, the CP of the different PSD weight fractions of corn flour showed a nonlinear relation when ST increases. The inset in Figure 3 exhibits the average scattering of CP for the 40, 60, and 80 mesh (black squares) and for the 30 and 100 mesh data (green triangles). A clear non-linear-curve tendency is observed there. These results are in agreement with the research published previously [8], which demonstrated that the incorporation of calcium into the maize kernel during the nixtamalization process follows a nonlinear process in proportion to the increase of ST. The experimental results from Figures 2 and 3 could be explained taking into account that water and calcium diffusion into the particles is limited, so that the diffusion of both materials must tend to zero for a long time; that is, a tendency to saturation should be observed in both cases.

### 4.3. Crystallinity Measurements

**Figure 4:**
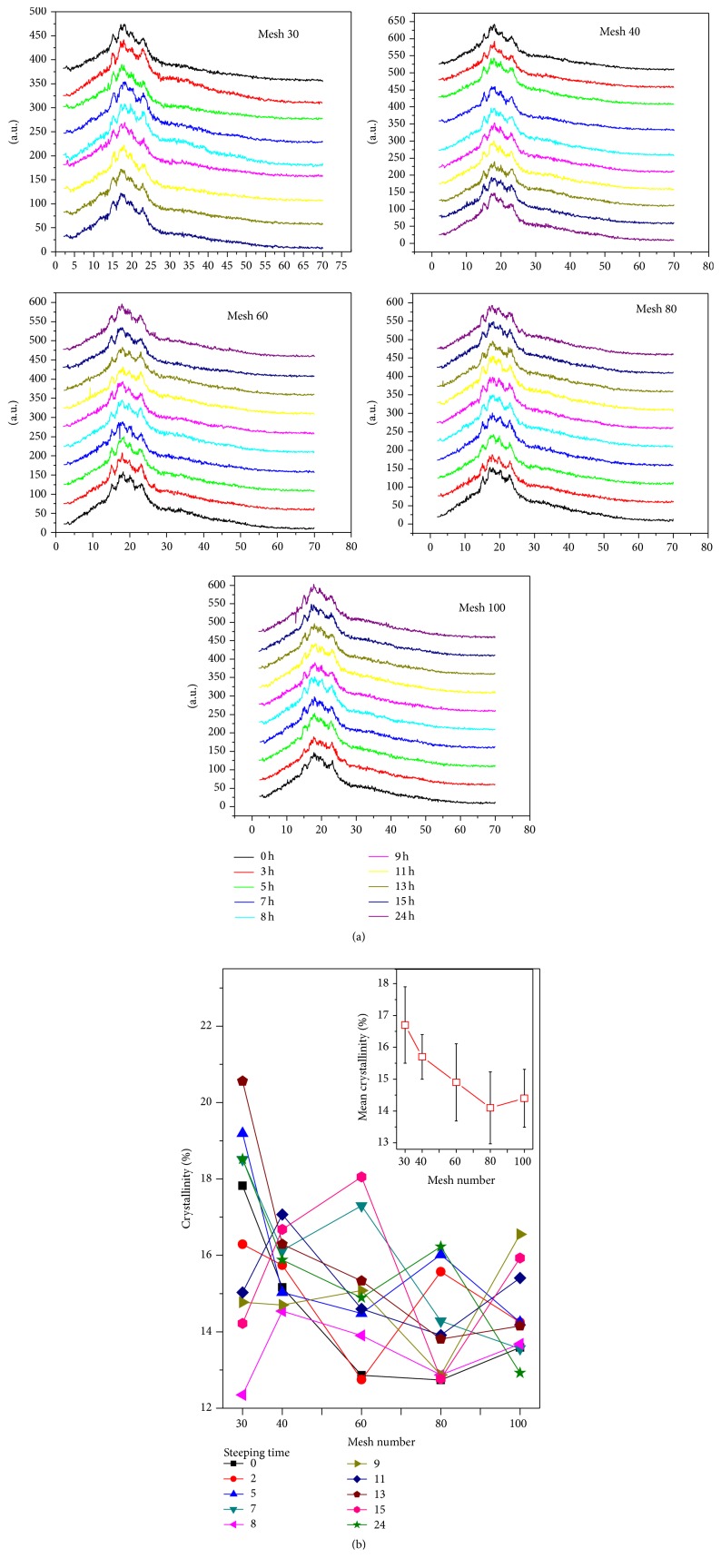
(a) X-ray diffractograms of the PSD samples for different ST. (b) The crystallinity of the PSD samples versus the mesh numbers chosen, for the different ST values, selected for the study. The inset displays the mean crystallinity, averaged on the twelve ST values, against the mesh number.

**Figure 5:**
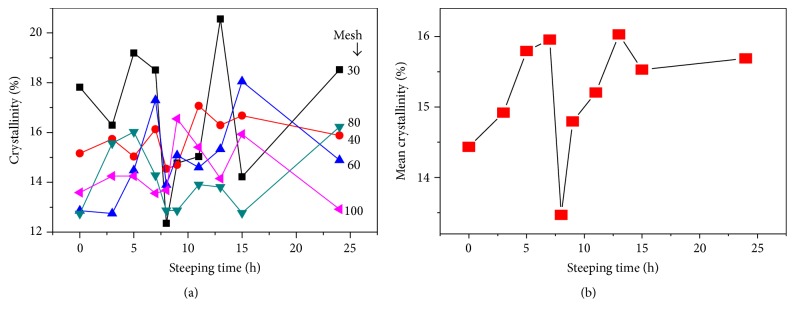
(a) Crystallinity of the PSD samples as a function ST for all the mesh numbers. (b) The mean value of crystallinity, averaged on the five mesh number values, against ST.

**Figure 6:**
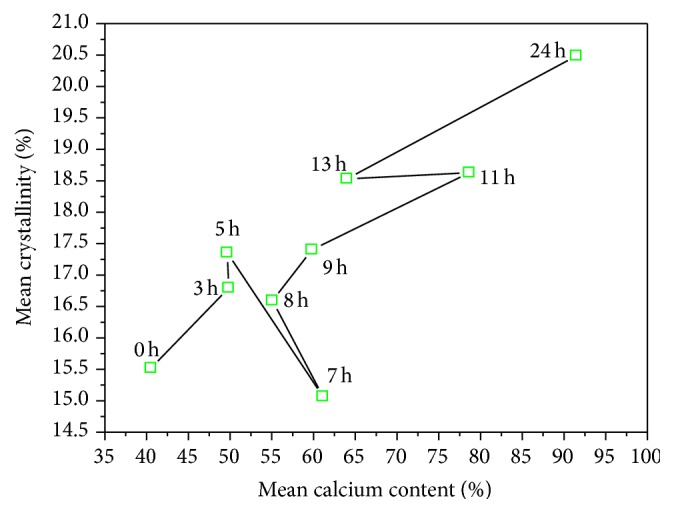
The crystallinity of samples versus the calcium content, both measurements averaged over the twelve ST values.

Figure 4(a) shows the X-ray diffractograms of PSD (30, 40, 60, 80, and 100) of corn flour as a function ST. The crystallinity percentages are calculated by normalizing the integrated crystallinity intensity to the integrated noncoherent intensity. The same procedure was used for all samples. The crystallinity percentage measurements as a function of ST and mesh number produces fluctuations (see Figures 4(b) and 5(b)) as in Figures 1 and 2, normally observed in measurements of PSDs. These values represent the average of three measurements per sample. The relative crystallinity variation of the corn flour as a function of the mesh number, processed at different STs, can be observed in Figure 4(b). The smaller particle size and the lower crystallinity are expected for materials with microns and submicron dimensions [12, 13]. Almost for all the graphs of Figure 4(b), this assertion is valid; the average of the crystallinity values for all the meshes confirms this fact, as is displayed in the inset of Figure 4. All the crystallinity data of the PSD samples are observed in the range from 12 to 21%. The change in the crystallinity of the PSD samples is due to the crystallization of starches, which in turn is promoted by the calcium diffusion process into the maize kernel during cooking and ST. The recrystallization of the starches occurs during the ST process [14]. Therefore, it is important to mention that the fluctuation in values of percentage of recrystallization of the starch in the maize depends on ST. In this study, the maximum value was observed for ST for 5, 7, and 13 h.  Figure 5(b) shows the crystallinity versus ST for the different meshes used. In Figure 5(b) the mean crystallinity values averaged from the data of the five meshes is displayed. Clearly, a critical point is observed at ST = 8 h. The critical point is related to the relative maximum of the calcium percentage around ST in the interval of 7–9 hours, as reported by other authors. At ST within this range, the pericarp is almost removed, entirely, which allows more diffusion of calcium into the corn kernel [15]. In the inset of Figure 3 the first relative maximum of CP at ST = 8 h can also be observed. The dependence of crystallinity on the calcium percentage is not linear, and it is not, after all, easy to predict. In our case, Figure 6 displays the mean crystallinity relative versus CP percentage averaged for all the meshes with the minimum at CP concentration in the 62% zone and ST within, in this case, the 7 h region. The minimum in the crystallinity indicates the greater gelatinization point of starch, where the crystallinity is significantly reduced. The graphs of Figures 5(b) and 6 could describe the crystallization-gelatinization-recrystallization process [16].

### 4.4. RVA Measurements

**Figure 7:**
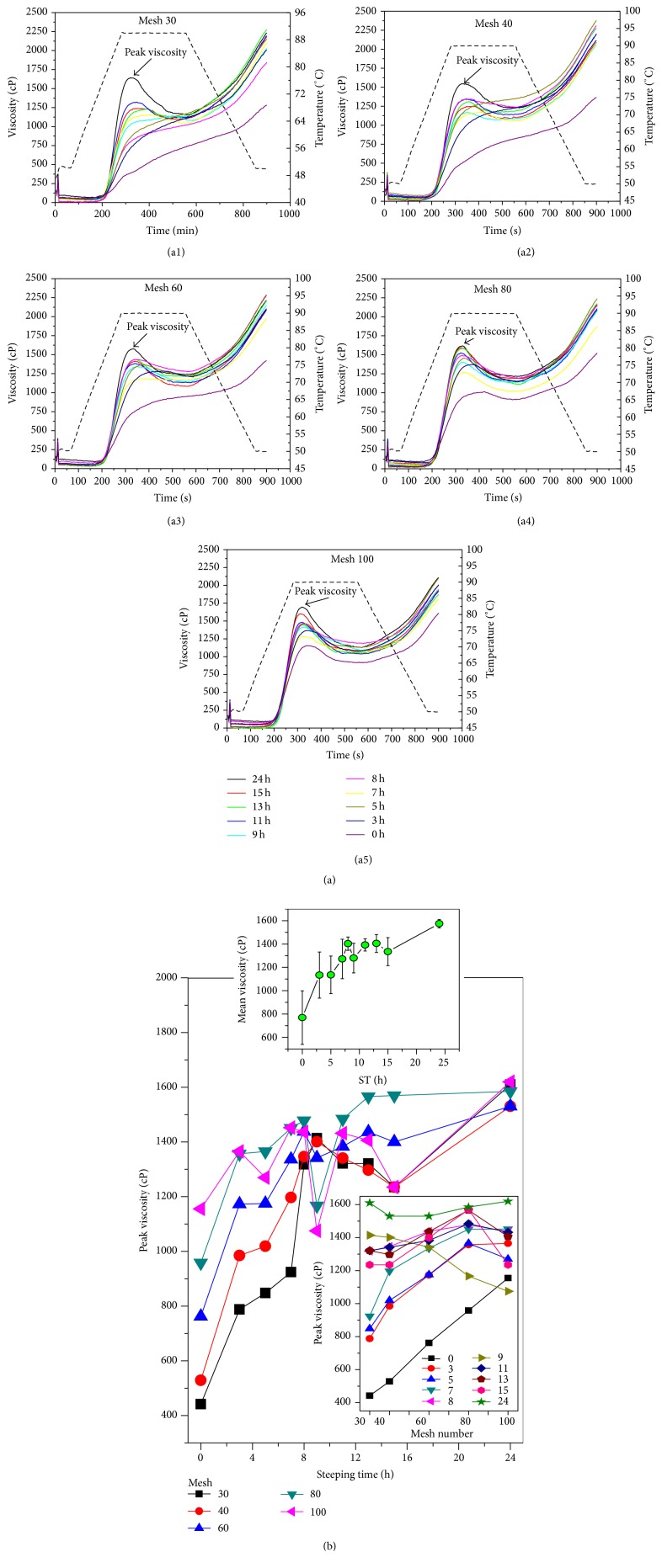
(a) RVA profiles of the instant corn flour for traditional nixtamalization process for samples at *t* = 0, 3, 5, 7, 8, 9, 11, 13, 15, and 24 h. (b) Peak viscosity measurements at 90°C displayed as a function of ST for all the mesh numbers. The inset on bottom exhibits the peak viscosity versus the mesh numbers for the twelve ST values. The inset above shows the mean viscosity, averaged on the five mesh numbers, as a function of ST.

Figure 7(a)((a1)–(a5)) showed the viscosity profiles of several PSD of the corn flour samples as a function of steeping time. The parameter read from the viscometer curves was the peak viscosity during the heating cycle. Several corn flour fractions showed significantly different RVA profiles characteristics. The peak viscosity and breakdown point were absent for the following parameters: mesh 30 and ST < 11 h; mesh 40 and ST < 7; mesh 60 and ST < 3 h; and meshes 80 and 100 for any ST. The samples that present viscosity peak and breakdown point have the following parameters: mesh 30 and ST > 11 h; mesh 40 and ST > 7 h; mesh 60 and ST > 3 h; and meshes 60 and 100 for ST ≥ 0 h.  Figure 7(b) shows the viscosity values at 90°C for the PSD samples of corn flour as a function of ST. These experimental values represent the average of three measurements per sample. The results show that the viscosity increases as ST rises, converging for all the PSDs to similar values for extended ST (24 h). Usually, the PV depends on the particle size; the larger (smaller) the particles the more minor (larger) the PV density [3]. In this work, the highest value of viscosity was observed for mesh 100 and any value of ST, and the minimum was observed for ST < 11 h and mesh 30 (see Figure 7(b)). For a ST of 24 h the five viscosity curves converge, probably because as the ST increases the starch is partially gelatinized due to the medium alkaline action [11]. In the inset of Figure 7(b) the mean crystallinity (averaged among the different screens used) versus ST indicates that a relative minimum of viscosity occurs at ST *≅* 8-9 h. The inset at the bottom of Figure 7(b) confirms the experimental fact that, in general, the larger (smaller) the particles size the more minor (larger) the viscosity.

## 5. Conclusions

The several flour corn fractions showed significantly different RVA profiles, PDS, and calcium and crystallinite percentages as function of ST. Therefore the* peak viscosity and breakdown point depended on calcium percentage, ST, and particle size*. The ST produces nonlinear changes in percentages of calcium, PDS, and crystallinity in the corn flour. For the corn flour obtained employing meshes 30, 40, and 60, the peak viscosity and breakdown point were observed as an effect of ST on the coarse particles.
